# Medullary-Sparing Antibiotic Cement Articulating Spacer Reduces the Rate of Mechanical Complications in Advanced Septic Hip Arthritis: A Retrospective Cohort Study

**DOI:** 10.3390/jpm14020162

**Published:** 2024-01-31

**Authors:** Chun-Yen Chen, Chin-Ping Lin, Chun-Hao Tsai, Hui-Yi Chen, Hsien-Te Chen, Tsung-Li Lin

**Affiliations:** 1 Department of Orthopedics, China Medical University Hospital, Taichung 40447, Taiwan; 028149@tool.caaumed.org.tw (C.-Y.C.); 024667@tool.caaumed.org.tw (C.-P.L.); 002326@tool.caaumed.org.tw (H.-T.C.); 2 Department of Sports Medicine, College of Health Care, China Medical University, Taichung 406040, Taiwan; 3 Department of Radiology, China Medical University Hospital, Taichung 40447, Taiwan; 007396@tool.caaumed.org.tw; 4 Graduate Institute of Biomedical Sciences, China Medical University, Taichung 40402, Taiwan

**Keywords:** advanced septic hip arthritis, articulating spacers, mechanical complications, medullary-sparing

## Abstract

Antibiotic cement articulating spacers eradicate infection during a two-stage revision for advanced septic hip arthritis (ASHA); however, mechanical complications have been reported. We hypothesized that the rate of mechanical complications would be lower in medullary-sparing (MS) than in non-medullary-sparing (n-MS) articulating spacers. A retrospective study of ASHA using n-MS or MS spacers was conducted between 1999 and 2019. The rate of mechanical complications and reoperation and risk factors for mechanical complications were analyzed. The cohort included 71 n-MS and 36 MS spacers. All patients were followed up for 2 years. The rate of spacer dislocation was lower in MS (0%) than in n-MS spacers (14.1%; *p* = 0.014). The reoperation rate for mechanical complications was lower in MS (0%) than in n-MS spacers (12.7%; *p* = 0.019). The rate of a diaphyseal stem during reimplantation was lower in MS (0%) than in n-MS spacers (19.4%; *p* = 0.002). The identified risk factors for n-MS spacer dislocation were postoperative under-restored femoral head diameter ≥3 mm, femoral offset ≥3 mm, and surgical volume (≤6 resection arthroplasties per year). Both spacers controlled infection. However, MS spacers had a lower spacer dislocation and reoperation rate and avoided the diaphyseal stem during reimplantation. We recommend using MS spacers to restore native femoral head diameter and femoral offset when ASHA is treated by surgeons with lower surgical volumes.

## 1. Introduction

Septic hip arthritis in adults occurs in 8 per 100,000 people/year but is time dependent with a propensity for irreversible joint damage [1,2,3]. Two-stage revision with antibiotic cement articulating spacers has gained popularity in treating patients who have advanced septic hip arthritis (ASHA), such as chronic infection, concomitant degenerative arthritis, or prior osteonecrosis of the femoral head, with great success in infection control and better functional outcomes [2,4,5,6].

The characteristics of bone-stock preservation can be broadly classified as medullary-sparing (MS) or non-medullary-sparing (n-MS) spacers [5,6,7,8,9,10]. Most spacers are n-MS, disturbing the proximal femoral marrow via the stem structure [5,6,7,8,9,10,11,12,13]. However, MS spacers preserve femoral bone by cutting the femoral neck alone [14,15].

The optimal construct has not yet been identified because of the broad range of spacer styles and relatively few case series in the literature. In addition, heterogeneous mechanical complications, such as spacer dislocation, spacer fracture, or peri-spacer fracture, have been reported, all of which are associated with n-MS spacers [8,10,11,13]. Moreover, these complications lead to reoperation, poor clinical outcomes, and prolonged treatment courses [8,11]. To the best of our knowledge, no studies have directly compared bone-stock preservation methods in articulating spacers. In this regard, a study comparing the results of n-MS and MS spacers is critical for two-stage revision for ASHA.

This study aimed to compare the rate of mechanical complications and reoperation during the interim stage and the rate of infection eradication after a two-stage revision with n-MS and MS spacers. We hypothesized fewer mechanical complications and reoperations with the use of MS spacers but no differences in the infection eradication rate. The risk factors for developing mechanical complications were also analyzed.

## 2. Materials and Methods

### 2.1. Patients

The local Institutional Review Board approved the study, and all participants provided written informed consent. The study population was derived from a retrospective database that included adult patients treated for ASHA who underwent a two-stage revision between January 1999 and December 2019 in a single center. The inclusion criteria were: (1) joint or blood culture positive infection, (2) pre-operative magnetic resonance imaging (MRI) of the hip, (3) antibiotic cement molded articulating spacer, and (4) minimum outpatient follow-up visits of 2 years. The exclusion criteria were: (1) Girdlestone hip; (2) static spacer; (3) handmade spacer; (4) previous osteosynthesis or arthroplasty of the affected or contralateral hip; (5) fungal or tuberculous hip; (6) infection spreading into femoral neck or canal rather than lesions confined to the hip joint (confirmed by pre-operative MRI); or (7) incomplete data including lack of pre-operative MRI or post-operative radiographs during follow-up and follow-up visits <2 years.

All included patients were categorized into two groups based on n-MS or MS1 spacers. Seventy-one n-MS spacers were implanted between January 1999 and December 2013 and 36 MS spacers were implanted after January 2014. Five trained arthroplasty surgeons performed all surgeries.

### 2.2. Surgical Technique

All spacers were made based on a 1:5 ratio of antibiotics/bone cement per package (CMW3 without adding gentamycin; DePuy Synthes, Warsaw, IN, USA) and were implanted via a routine anterolateral approach under general anesthesia. If a specific culture organism was identified preoperatively, directed antibiotic choices were made; if it was an unknown organism, 4 g of vancomycin and 4 g of ceftazidime per 40 g of bone cement were used [16].

**Figure 1:**
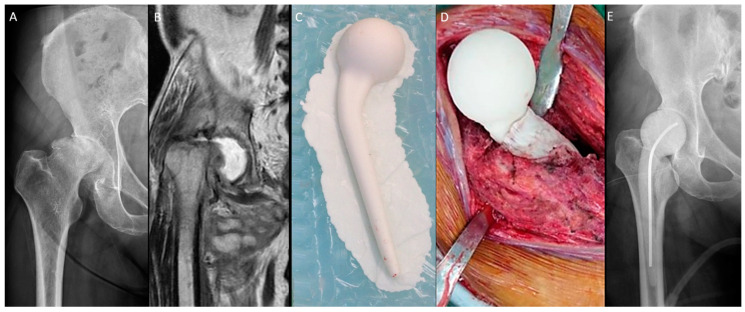
Non-medullary-sparing (n-MS) spacers. (**A**) Pre-operative radiograph showing cartilage destruction with the subchondral collapse of the femoral head, (**B**) pre-operative MRI showing diffuse fluid accumulation within the joint, (**C**) fabrication of the n-MS spacer using a silicone mold, (**D**) the hip joint with the n-MS spacer implanted, and (**E**) post-operative radiograph with the n-MS spacer in situ.

The n-MS spacer (Figure 1) was fabricated with an intraoperative silicone mold. Four sizes of the spacer trials and molds were available (Supplementary Table S1). After dislocation, the femoral neck was osteotomized with one finger width left above the lesser trochanter. The size of the femoral head was measured, and then a roughly compatible spacer size was determined. The acetabulum was prepared with a reamer to remove residual cartilage until oozing and 1 mm greater than the selected spacer head size. Then, the femoral medulla was prepared with a broach to fit the corresponding size of the spacer stem. Molding of the spacer and bony preparation were performed simultaneously. The spacer was prepared with a curved 3.0 mm Kirschner wire or 10.0 mm stainless-steel rod as the central endoskeleton until the cement cured. After adequate debridement, the spacer was cemented proximally within the medulla with a neutral version [17]. The hip was reduced, stability was assessed, and the capsule was repaired.

**Figure 2:**
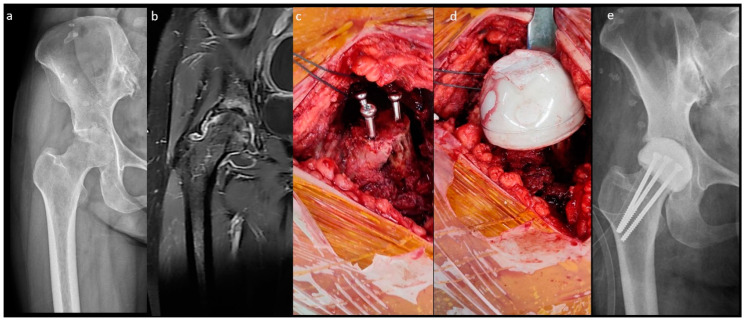
Medullary-sparing (MS) spacers. (**a**) Pre-operative radiograph showing diffuse cartilage destruction, (**b**) pre-operative MRI showing diffuse fluid accumulation within the joint, (**c**) three endoskeletal screws inserted to the femoral neck, (**d**) fabrication of the MS spacer using the bulb-shaped mold, and (**e**) post-operative radiograph with the MS spacer in situ.

The fabrication of the MS spacer is shown in Figure 2. The femoral neck was osteotomized over the sub-capital level. After the same acetabular procedure was performed as in the n-MS spacer, three parallel 4.5 mm cancellous screws were inserted into the remaining femoral neck as endoskeletons for the spacer. Then, cement was attached to the screws with a personalized bulb-shaped irrigation syringe as a mold to restore the corresponding height and diameter of the original femoral head [18]. Finally, the hip was reduced with capsular repair, as in the n-MS spacer.

### 2.3. Post-Operative Protocol

Post-operative weight-bearing was similar for both spacers: toe-touch with crutches. Intravenous antibiotics were administered according to the susceptibilities of each microorganism for at least 4 weeks, until a progressive decline in C-reactive protein (CRP) and erythrocyte sedimentation rate (ESR) levels. Oral suppressive antibiotics were continued for at least 4 weeks until CRP and ESR levels normalized to less than 1 milligram per liter and 20 mm per hour, respectively. Radiographic evaluation of the hip, including anterior–posterior and lateral views, was conducted post-operatively and every subsequent month, pre-reimplantation, or at any time the patient experienced severe hip pain.

Criteria for reimplantation included CRP and ESR values within normal limits, and negative signs of infection after at least 2 weeks of antibiotic holiday.

### 2.4. Evaluation

**Figure 3:**
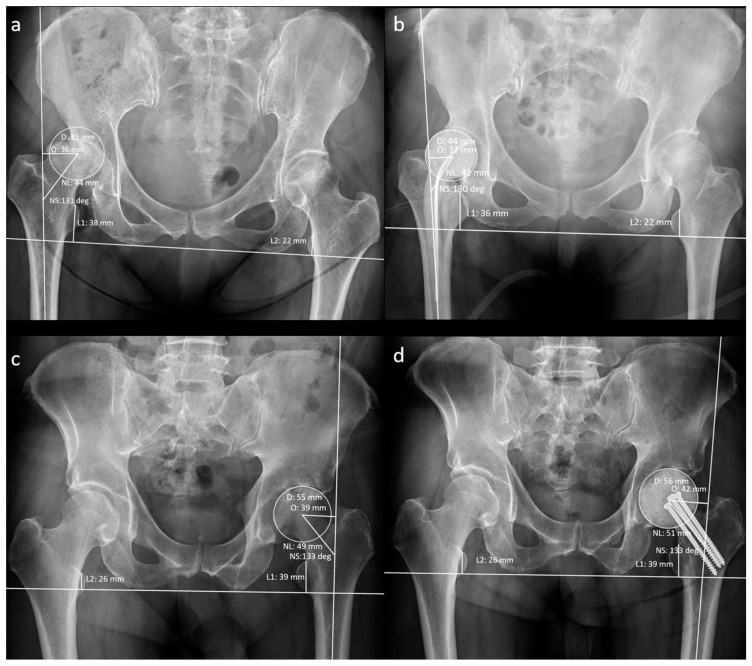
Measurement of radiographic hip geometrical parameters. (**a**) Pre-operative n-MS hip, (**b**) post-operative n-MS hip, (**c**) pre-operative MS hip, and (**d**) post-operative MS hip. Δhip parameters: femoral head diameter difference (post-operative D–pre-operative D), femoral neck length difference (post-operative NL–pre-operative NL), neck–shaft angle difference (post-operative NS–preoperative NS), femoral offset difference (post-operative O–pre-operative O), and leg-length discrepancy difference (post-operative L1–L2)–(pre-operative L1–L2).

The patients’ demographic data at the time of resection arthroplasty (RA) were recorded. A musculoskeletal radiologist recorded the hip geometrical parameters using preoperative and post-operative anteroposterior radiographs (Figure 3).

The following post-operative radiographic findings were recorded as spacer mechanical complications using the INFINITT Picture Archiving and Communications System: (1) spacer dislocation, (2) spacer fracture (including endoskeleton broken), or (3) peri-spacer fracture. A musculoskeletal radiologist and two arthroplasty surgeons assessed and recorded all the radiographic data.

Information, such as reoperation for spacer mechanical complications or reinfection such as debridement, arthrotomy, or spacer exchange, was recorded during the interim stage.

Usage of bone graft and stem type during reimplantation were recorded. Infection eradication was defined using the Delphi criteria after reimplantation [19]. The infection rate was recorded annually and at the most recent evaluation. All the above data were reviewed by one author blinded to the surgeries.

### 2.5. Statistical Analysis

Descriptive statistics are presented as means and 95% confidence intervals for continuous variables and as counts and percentages for categorical variables. Differences between the two groups were assessed using Student’s *t*-test for continuous variables and Fisher’s exact test for categorical variables. The reliability of spacer mechanical complications was examined by the intraclass correlation coefficient. Univariate and multivariate logistic regression models were performed to assess the association of covariates with the spacer mechanical complication risk. Statistical significance was set at *p* < 0.05. Statistical analyses were performed using SPSS for Windows version 24 (IBM Corp., Armonk, NY, USA).

## 3. Results

**Figure 4:**
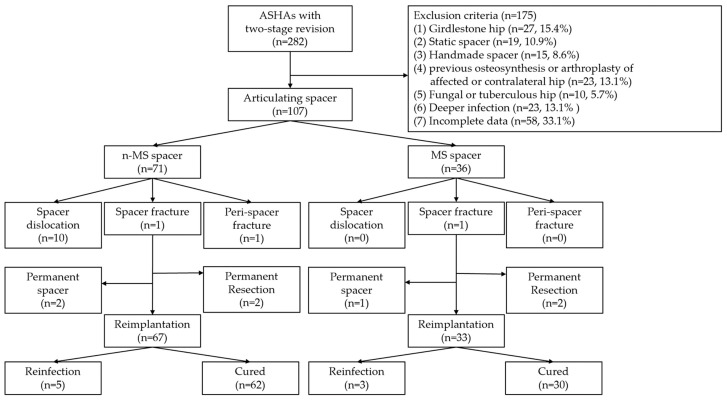
STROBE flowchart detailing the design of the study. STROBE, Strengthening the Reporting of Observational Studies in Epidemiology; ASHA, advanced septic hip arthritis; n-MS, non-medullary sparing; MS, medullary sparing.

In total, 107 patients were included and followed up for 64.7 months. The diagnosis of ASHA was recalcitrant to antibiotics and serial debridement in 51 (47.7%), chronic infection in 28 (26.2%), concomitant degenerative arthritis in 15 (14.0%), and concomitant osteonecrosis of the femoral head in 13 (12.1%) patients. Twenty-two (20.6%) and 28 (26.2%) patients underwent arthrotomy and percutaneous abscess drainage, respectively, at other institutions before visiting our clinic. For each included patient, one hip was considered. There were 71 and 36 n-MS and MS spacers, respectively. The STROBE flow chart detailing the study design is shown in Figure 4. The baseline determinants and outcomes between the included and excluded cohorts with incomplete data are presented in Supplementary Tables S2 and S3. There were no significant differences in demographic data between individuals receiving n-MS or MS spacers, except during the follow-up period (Table 1).

**Figure 5:**
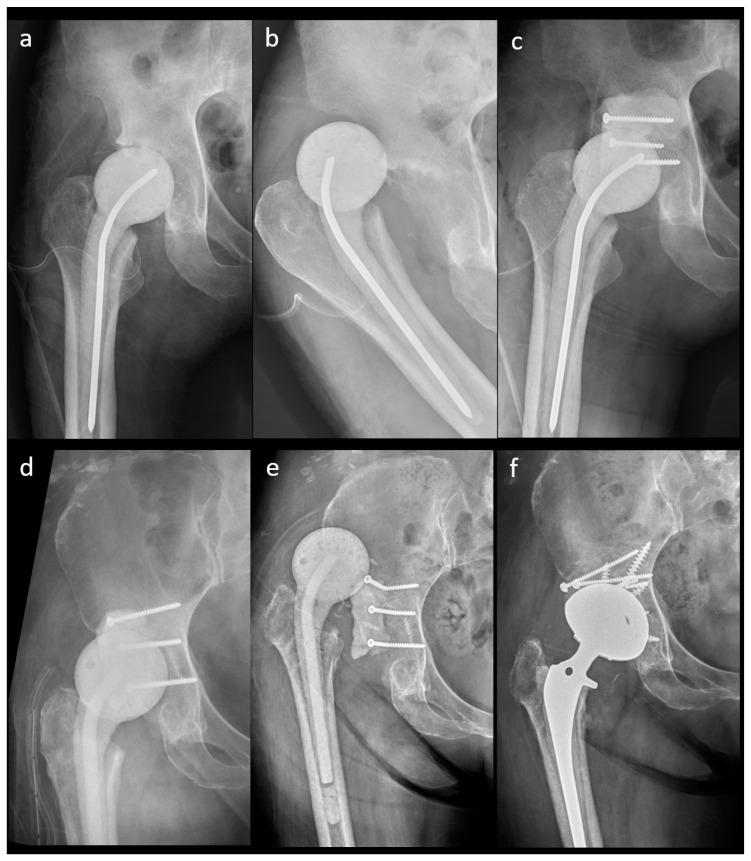
A 41-year-old female patient with an n-MS spacer: (**a**) post-operative radiographs, (**b**) posterior dislocation 2 weeks after spacer insertion, and (**c**) open reduction combined with temporary cement tectoplasty. A 68-year-old male patient with an n-MS spacer: (**d**) open reduction combined with tectoplasty after posterior dislocation 4 weeks after spacer insertion, (**e**) recurrent dislocation after tectoplasty, and (**f**) acute reimplantation with bone grafting and cemented stem after the dislocation event and free of complications.

The outcomes of the n-MS and MS spacers are shown in Table 2. The intraclass correlation coefficient of spacer mechanical complications was 0.923 (range: 0.901–0.973, *p* < 0.001). The rate of spacer dislocation was significantly lower in MS (n = 0) than in n-MS (n = 10) spacers. The characteristics of joint dislocation were 100.0% nontraumatic posteriorly and 90.0% with early failure ≤3 weeks after n-MS spacer insertion (Table 3). Among 10 dislocated spacers, one underwent close reduction, four underwent open reduction with temporary cement tectoplasty (TCT) (Figure 5a–c), and four underwent spacer exchange with TCT. All patients were advised to use abduction braces after relocation and were free of dislocation. However, one underwent acute total hip arthroplasty (THA) without complications because of recurrent dislocation after 2 weeks of TCT (Figure 5d–f). Additionally, spacer retention was chosen for one patient because he was medically unfit for further surgery. One hip underwent permanent resection due to concomitant recurrent infection. The reoperation rate for spacer mechanical complications was significantly lower in MS than in n-MS spacers due to dislocated joints.

During reimplantation, n-MS spacers were associated with an increased requirement for a diaphyseal stem, such as a Wagner self-locking revision stem (Zimmer, Warsaw, IN, USA). However, the infection eradication rate was comparable between both spacers (Table 2).

Supplementary Table S4 presents univariate risk factors for n-MS spacer dislocation. On multivariate logistic regression analysis, post-operative under-restored femoral head diameter ≥ 3 mm and femoral offset ≥ 3 mm, and surgical volume ≤ 6 RAs/year were identified as independent risk factors for n-MS spacer dislocation (Table 4).

## 4. Discussion

The results demonstrated that MS spacers carry a lower risk of spacer dislocation requiring fewer reoperations in the interim stage, and diaphyseal stems during reimplantation can be avoided. Moreover, the identified risk factors for spacer dislocation were post-operative under-restored femoral head diameter, femoral offset, and lower surgical volume; both spacers controlled infection.

In the two-stage revision of ASHA, the n-MS spacer, originally designed for prosthetic hip infection (PHI), contained a stem structure and could improve functional outcomes, reduce pain, and control infection [5,6,7,8,9,11,12,13]. Despite the success of articulating spacers, spacer dislocation is the most frequently reported mechanical complication (approximately 17%), leading to a higher rate of complex acetabular reconstruction, constrained liners, and dislocation after reimplantation [20]. Decreased leg length and offset, inadequate head/neck ratio, head/acetabular diameter mismatch, excess spacer anteversion, and limited head sizing and offset options of spacers may contribute to a high spacer dislocation rate in PHI [17,21,22,23]. In this study, the spacer dislocation rate was 14.1% for n-MS spacers. These dislocations were all atraumatic posteriorly, with 90.0% early dislocation ≤ 3 weeks after spacer insertion.

In the setting of infection spreading into femoral neck or canal rather than lesions confined of the hip joint in ASHA, insertion of a medullary spacer is reasonable [24]. In contrast to PHI, the acetabulum, proximal femur, and musculature remain intact in ASHA; however, it is difficult to restore the native hip biomechanical parameters with the current four sizes of n-MS spacers, which have limited head diameter, neck length, neck–shaft angle, and femoral offset. In the past, dislocations were attributed to anterior impingement when transitioning position and to subsequent posterior instability, and most patients were free of dislocation after TCT [25]. All studies available on PubMed until the present date on mechanical complications of cement articulating spacers in ASHA are summarized in Table 5. In the current study, n-MS spacers showed a higher complication rate than that in other studies, which might have been related to a higher frequency of follow-up. All images were rigorously evaluated by one musculoskeletal radiologist and two arthroplasty surgeons.

Shen et al. described the use of MS spacers in ASHA whereby the cement head was fixed with endoskeletal pins in the femoral neck without opening the femoral canal [14]. Cho et al. used screws to improve the fixation of the cement head [15]. Both MS spacers were effective for infection eradication and preserving the femoral medullary canal and were free of mechanical complications. With the current MS spacers, the preservation of the native neck length, neck–shaft angle, femoral offset, and anteversion could be achieved by osteotomy at the sub-capital level and preservation of the original neck. MS spacers could retain the native hip biomechanical parameters to decrease the spacer dislocation.

Limited head sizing in preformed or molded spacers may contribute to a relatively high dislocation rate [21,26,27]. Barreira et al. reported that an undersized head could be a risk factor for spacer dislocation, and TCT was advised if a larger head was unavailable [17]. In the current four sizes of n-MS spacers, all dislocated spacers were under-restored with a head diameter ≥3 mm. There was no dislocation in MS spacers, and this might be because the personalized bulb-shaped molded head could precisely restore the native height and diameter.

In THA, Heckmann et al. found that under-restored hip offset ≥3 mm was markedly associated with dislocation [28]. The biomechanical advantage of hip offset restoration decreased the risk of bony impingement [29]. Jones et al. reported that reduced femoral offset >5 mm carried a high risk of spacer dislocation [22]. The reduction in abductor muscle tension resulting from a decrease in femoral offset was also an important cause of spacer dislocation [13,22,30]. In our study, all dislocated spacers were n-MS type, and the under-restored femoral offset ≥3 mm could increase dislocation risk. The surgeon must pay attention to restoring a patient’s native offset, including using a high-offset or lateralized spacer.

In a propensity-matched cohort study, there was an increased risk of dislocation and early revision with surgical volume ≤ 35 THAs/year [31]. Moreover, according to the American Joint Replacement Registry, low surgical volume was associated with early dislocation following THA [32] and increased surgical volume was associated with lower THA dislocation rates [33,34]. No prior study has analyzed the effects of surgical volume and RA. In the current study, surgical volume ≤6 RAs/year was associated with high spacer dislocation rate. The low surgical volume of RAs might result in mal-sizing spacers [35], mal-anteversion, mal-depth insertion, and inadequate spacer fixation, which increase dislocation risk.

Both the MS and n-MS spacers controlled infection comparably. However, n-MS spacers were associated with an increased requirement for a diaphyseal stem during reimplantation. Hence, there is a possibility that disrupting the healthy marrow may impede the subsequent biological metaphyseal fixation of the femoral stem [15]. Conversely, preserving the proximal femoral marrow of the MS spacer could minimize the usage of the diaphyseal stem [14,15]. In reimplantation, the MS hip was similar to the sub-capital femoral neck fracture with an intact proximal femur and could easily be managed with metaphyseal or cemented stem, avoiding the use of the diaphyseal stem for passing the violated unhealthy proximal femur in n-MS, such as in PHI.

This study had some limitations. First, this was a retrospective cohort study involving multiple surgeons; n-MS spacers were implanted in an earlier study period, which might have resulted in more spacer complications due to the learning curve. Second, the underestimation of spacer complication rates for MS spacers might be have been due to the smaller sample size and shorter follow-up period. However, most spacer dislocations were early failures after insertion, which minimized this bias. Third, the lack of pre- and post-operative computed tomography (CT) data prevented the accurate measurement of hip parameters [36]. Preoperative CT of the hip is needed to personalize the n-MS spacer or create more mold sizes, especially for racial and sexual anatomical variations [37]. Finally, the limited sizing of the n-MS spacer was insufficient for every hip morphology.

## 5. Conclusions

This is the first retrospective cohort study to compare MS and n-MS spacers in a two-stage revision of ASHA. Both spacers can control infection; however, MS spacers had a lower spacer dislocation and reoperation rate and avoided the diaphyseal stem during reimplantation. We recommended using MS spacers when ASHA is treated by surgeons with lower surgical volumes to restore native femoral head diameter and femoral offset. More extensive prospective studies are required.

## Figures and Tables

**Table 1:** Demographic data of patients with n-MS and MS spacers.

Variables	Spacer Type		*p*-Value
	n-MS (n = 71)	MS (n = 36)	
Age, years (95% CI)	56.3 (38–68)	58.6 (41–69)	0.662
Female, n (%)	34 (47.9)	19 (52.8)	0.461
Body mass index, kg/m^2^ (95% CI)	24.9 (20.7–31.4)	23.8 (21.2–29.6)	0.655
Right laterality, n (%)	38 (53.5)	17 (47.2)	0.413
Current/ex-smokers, n (%)	29 (40.8)	13 (36.1)	0.560
Insurance status			
Insured, n (%)	64 (90.1)	32 (88.9)	0.883
Any form of Medicaid, n (%)	7 (9.9)	4 (11.1)	0.719
Uninsured, n (%)	0 (0.0)	0 (0.0)	0.999
Socioeconomic status			
Low, n (%)	33 (46.5)	16 (44.4)	0.712
Middle, n (%)	21 (29.6)	11 (30.6)	0.725
High, n (%)	17 (23.9)	9 (25.0)	0.616
Charlson comorbidity index			
0–2, n (%)	39 (54.9)	18 (50.0)	0.437
3+, n (%)	32 (45.1)	18 (50.0)	0.408
McPherson host grade			
Uncompromised, n (%)	14 (19.7)	8 (22.2)	0.542
Compromised, n (%)	20 (28.2)	11 (30.6)	0.616
Significantly compromised, n (%)	37 (52.1)	17 (47.2)	0.478
Microorganisms			
Gram-positive species, n (%)	42 (59.1)	20 (55.6)	0.544
Gram-negative species, n (%)	23 (32.4)	13 (36.1)	0.583
Polymicrobial, n (%)	6 (8.5)	3 (8.3)	0.832
Pre-operative acetabular bone defects			
Paprosky type I, n (%)	61 (85.9)	29 (80.6)	0.626
Paprosky type II, n (%)	10 (14.1)	7 (19.4)	0.703
Paprosky type III, n (%)	0 (0)	0 (0)	0.999
Pre-operative hip parameters			
Femoral head diameter, mm (95% CI)	49.6 (41–60)	48.3 (42–57)	0.693
Femoral neck length, mm (95% CI)	44.2 (38–49)	43.8 (40–47)	0.748
Neck–shaft angle, degree (95% CI)	132.6 (124–145)	133.8 (130–148)	0.377
Femoral offset, mm (95% CI)	37.7 (30–43)	37.9 (32–44)	0.402
Leg-length discrepancy, mm (95% CI)	−8.7 (−18 to −1)	−9.3 (−17 to −2)	0.631
Underwent arthrotomy history, n (%) (22)	15 (21.1)	7 (19.4)	0.568
Surgical time, min (95% CI)	151.6 (109–179)	153.9 (115–185)	0.564
Surgical blood loss, mL (95% CI)	643.7 (300–910)	666.8 (320–950)	0.757
Post-operative hip parameters			
Femoral head diameter, mm (95% CI)	47.6 (44–56)	48.5 (42–59)	0.793
Femoral neck length, mm (95% CI)	45.2 (42–46)	43.7 (40–47)	0.511
Neck–shaft angle, degree (95% CI)	130.0 (130–130)	133.8 (130–148)	0.272
Femoral offset, mm (95% CI)	34.7 (32–36)	37.7 (31–43)	0.242
Leg length discrepancy, mm (95% CI)	−2.7 (−6–0)	−0.3 (−2–2)	0.136
Interim period, weeks (95% CI)	16.1 (10–18) ^a^	14.7 (9–16) ^b^	0.293
Follow-up period, months (95% CI)	98.2 (61–148) ^a^	39.5 (26–59) ^b^	<0.001

n-MS, non-medullary sparing; MS, medullary sparing; CI, confidence interval. ^a^ Two hips with permanent spacers and two hips with permanent resection were excluded. ^b^ One hip with permanent spacers and two hips with permanent resection were excluded.

**Table 2:** Outcomes of patients with n-MS and MS spacers.

Parameters	Spacer Type		*p*-Value
	n-MS (n = 71)	MS (n = 36)	
Spacer mechanical complications			
Spacer dislocation, n (%)	10 (14.1)	0 (0.0)	0.014
Spacer fracture, n (%)	1 (1.4)	1 (2.8)	0.247
Peri-spacer fracture, n (%)	1 (1.4)	0 (0)	0.813
Reoperation			
For spacer mechanical complications, n (%)	9 (12.7)	0 (0.0)	0.019
For reinfection, n (%)	8 (11.3)	4 (11.1)	0.530
During reimplantation			
Bone graft used in the acetabulum, n (%)	8 (11.9) ^a^	3 (9.1) ^b^	0.585
Uncemented metaphyseal stem, n (%)	46 (68.7) ^a^	30 (90.9) ^b^	0.004
Uncemented diaphyseal stem, n (%)	13 (19.4) ^a^	0 (0.0) ^b^	0.002
Cemented stem, n (%)	8 (11.9) ^a^	3 (9.1) ^b^	0.376
Dislocation after reimplantation, n (%)	6 (8.9) ^a^	2 (6.1) ^b^	0.251
Infection eradication after reimplantation, n (%)	62 (92.5) ^a^	30 (90.9) ^b^	0.872

n-MS, non-medullary sparing; MS, medullary sparing. ^a^ Two hips with permanent spacers and two hips with permanent resection were excluded. ^b^ One hip with permanent spacers and two hips with permanent resection were excluded.

**Table 3:** Details of patients with spacer dislocation in n-MS spacers.

Patient No.	Age (Years)	Sex	Mechanism	Timing Post Spacer (Weeks)	Radiographic Finding	Intervention
1	61	F	Nontraumatic	1	PD	CR
2	41	F	Nontraumatic	2	PD	OR with TCT
3	68	M	Nontraumatic	4	PD	OR with TCT ^a^
4	57	F	Nontraumatic	2	PD	OR with TCT
5	63	F	Nontraumatic	3	PD	OR with TCT
6	78	M	Nontraumatic	2	PD	OR with n-MS spacer exchange and TCT ^b^
7	53	F	Nontraumatic	2	PD	OR with n-MS spacer exchange and TCT
8	63	M	Nontraumatic	3	PD	OR with n-MS spacer exchange and TCT
9	56	F	Nontraumatic	1	PD	OR with n-MS spacer exchange and TCT
10	77	M	Nontraumatic	2	PD	Permanent resection ^c^

n-MS, non-medullary sparing; No., number.; PD, posterior dislocation; CR, close reduction; OR, open reduction; TCT, temporary cement tectoplasty. ^a^ Recurrent dislocation after TCT. ^b^ Permanent spacers. ^c^ Concomitant with recurrent infection.

**Table 4:** Multivariate analysis for risk factors associated with spacer dislocation in n-MS spacers.

Variables	Adjusted Odds Ratio (95% CI)	*p*-Value
Socioeconomic status, middle	2.53 (0.36–9.41)	0.092
McPherson host grade, uncompromised	2.91 (1.20–11.0)	0.071
Pre-operative acetabular bone defects, Paprosky type II	3.86 (1.08–12.3)	0.068
Post-operative femoral head diameter ≤ 44 mm	3.07 (0.97–11.6)	0.077
Post-operative under-restored femoral head diameter (≥3 mm)	11.6 (2.52–72.6)	0.002
Post-operative under-restored femoral offset (≥3 mm)	9.16 (1.60–63.7)	0.007
Surgical volume ≤ 6 resection arthroplasties/year	6.92 (1.57–58.4)	0.019

n-MS, non-medullary sparing.

**Table 5:** Summary of literature based on mechanical complications.

First Author	Year	Spacer Type	Number	Spacer Dislocation, n (%)	Spacer Fracture, n (%)	Peri-Spacer Fracture, n (%)	Infection Eradication Rate (%)
Kelm [11]	2009	n-MS	10	1 (10.0)	0 (0.0)	0 (0.0)	90.0
Huang [9]	2010	n-MS	15	0 (0.0)	0 (0.0)	2 (13.3)	100.0
Fleck [6]	2011	n-MS	14	0 (0.0)	0 (0.0)	0 (0.0)	100.0
Romanò [7]	2011	n-MS	20	2 (10.0)	0 (0.0)	0 (0.0)	95.0
Anagnostakos [8]	2016	n-MS	22	2 (9.1)	3 (13.6)	0 (0.0)	87.0
Russo [13]	2021	n-MS	25	2 (8.0)	0 (0.0)	0 (0.0)	92.0
Shen [14]	2013	MS	5	0 (0.0)	0 (0.0)	0 (0.0)	100.0
Cho [15]	2018	MS	10	0 (0.0)	0 (0.0)	0 (0.0)	100.0
Chen	Current study	n-MS	71	10 (14.1)	1 (1.4)	1 (1.4)	92.5

n-MS, non-medullary sparing; MS, medullary sparing.

## Data Availability

The authors declare that all data supporting the findings of this study are available within the article and its supplementary information files.

## Supplementary Materials

The following supporting information can be downloaded at: https://www.mdpi.com/article/10.3390/jpm14020162/s1, Table S1: Corresponding design of n-MS hip spacers; Table S2: Baseline determinants of the included and excluded cohort; Table S3: Outcomes between the included cohort and excluded cohort with incomplete data; Table S4: Univariate analysis of risk factors associated with spacer dislocation in n-MS spacers.
