# Supplementary material for: Medullary-Sparing Antibiotic Cement Articulating Spacer Reduces the Rate of Mechanical Complications in Advanced Septic Hip Arthritis: A Retrospective Cohort Study
Source: J Pers Med. 2024 Jan 31;14(2):162. doi: 10.3390/jpm14020162 (PMC10890418; doi:10.3390/jpm14020162)
Supplement: Supplementary file 1 [file jpm-14-00162-s001.zip › jpm-2779969-supplementary.pdf]

Supplementary Material

Supplementary Table S1. Corresponding design of n-MS hip spacers

Spacer (No.)	Femoral head diameter (mm)	Femoral neck length (mm)	Neck shaft angle (degree)	Femoral offset (mm)	Stem DxL (mm)
1	44	42	130	32	10 × 130
2	50	44	130	34	11× 130
3	56	46	130	36	12 × 130
4	62	48	130	38	13 × 130

n-MS, non-medullary sparing; No., number; D, diameter; L, length; mm, millimeter

Supplementary Table S2. Baseline determinants of the included and excluded cohort

Variables	Included cohort (n=107)	Excluded cohorts for incomplete data (n=58)	P-value
Age, years (95% CI)	57.7 (39–68)	59.2 (43–64)	0.538
Female, n (%)	53 (49.5)	27 (46.6)	0.331
Body mass index, kg/m^2 (95% CI)	24.1 (21.3–30.8)	24.4 (20.6–28.1)	0.704
Right laterality, n (%)	55 (51.4)	31 (53.4)	0.516
Current/ex-smokers, n (%)	42 (39.3)	21 (36.2)	0.430
Insurance status			
Insured, n (%)	96 (89.7)	53 (91.4)	0.646
Any form of Medicaid, n (%)	11 (10.3)	5 (8.6)	0.523
Uninsured, n (%)	0 (0.0)	0 (0.0)	0.999
Socioeconomic status			
Low, n (%)	49 (45.8)	25 (43.1)	0.608
Middle, n (%)	32 (29.9)	19 (32.8)	0.416
High, n (%)	26 (24.3)	14 (24.1)	0.830
Charlson comorbidity index			
0–2, n (%)	57 (53.3)	32 (55.2)	0.691
3+, n (%)	50 (46.7)	26 (44.8)	0.556
McPherson host grade			

Uncompromised, n (%)	22 (20.6)	13 (22.4)	0.638
Compromised, n (%)	31 (28.9)	18 (31.0)	0.519
Significantly compromised, n (%)	54 (50.5)	27 (46.6)	0.475
Microorganisms			
Gram-positive species, n (%)	62 (57.9)	33 (56.9)	0.773
Gram-negative species, n (%)	36 (33.6)	23 (39.7)	0.450
Polymicrobial, n (%)	9 (8.4)	2 (3.4)	0.468
Preoperative acetabular bone defects			
Paprosky type I, n (%)	90 (84.1)	50 (86.2)	0.601
Paprosky type II, n (%)	17 (15.9)	8 (13.8)	0.750
Paprosky type III, n (%)	0 (0)	0 (0)	0.999
Preoperative hip parameters			
Femoral head diameter, mm (95% CI)	48.8 (42–58)	47.5 (40–59)	0.804
Femoral neck length, mm (95% CI)	43.7 (37–47)	42.9 (38–46)	0.686
Neck-shaft angle, degree (95% CI)	133.0 (127–144)	132.7 (129–146)	0.422
Femoral offset, mm (95% CI)	37.5 (31–44)	37.7 (30–42)	0.673
Leg-length discrepancy, mm (95% CI)	-8.2 (-16 to -2)	-8.6 (-13 to -3)	0.706
Underwent arthrotomy history, n (%) (22)	22 (20.6)	13 (22.4)	0.609
Surgical time, min (95% CI)	152.1 (111–182)	150.7 (113–180)	0.411

Surgical blood loss, mL (95% CI)	651.9 (310–930)	642.0 (300–900)	0.826
Postoperative hip parameters			
Femoral head diameter, mm (95% CI)	48.1 (43–58)	47.5 (41–56)	0.640
Femoral neck length, mm (95% CI)	44.6 (41–46)	44.7 (42–46)	0.911
Neck-shaft angle, degree (95% CI)	132.3 (126–143)	134.1 (129–145)	0.439
Femoral offset, mm (95% CI)	36.1 (31–39)	36.6 (30–41)	0.708
Leg length discrepancy, mm (95% CI)	-2.1 (-5–0)	-1.3 (-4 to -1)	0.337
Interim period, weeks (95% CI)	15.3 (11–17) [a]	13.9 (10–15) [b]	0.304
Follow-up period, months (95% CI)	71.1 (31–144) [a]	29.8 (6–123) [c]	**<0.001**[d]

CI, confidence interval

[a]n=100 after excluding three hips with permanent spacers and four hips with permanent resection

[b]n=56 after excluding two hips with permanent spacers

[c]n=31 after excluding 27 hips with follow-up <2 years

[d]Bold font indicates significant differences (P<0.05).

Supplementary Table S3. Outcomes between the included cohort and excluded cohort with incomplete data

Variables	Included cohort (n=107)	Excluded cohorts for incomplete data (n=58)	P-value
Spacer mechanical complications			
Spacer dislocation, n (%)	10 (9.3)	5 (10.2) [a]	0.651
Spacer fracture, n (%)	2 (1.9)	1 (2.0) [a]	0.847
Peri-spacer fracture, n (%)	1 (0.9)	1 (2.0) [a]	0.446
Reoperation			
For spacer mechanical complications, n (%)	9 (8.4)	5 (10.2) [a]	0.403
For reinfection, n (%)	12 (11.2)	6 (10.3)	0.701
During reimplantation			
The bone graft used in the acetabulum, n (%)	11 (11.0) [b]	6 (10.7) [c]	0.773
Uncemented metaphyseal stem, n (%)	76 (76.0) [b]	41 (73.2) [c]	0.608
Uncemented diaphyseal stem, n (%)	13 (13.0) [b]	8 (14.3) [c]	0.694
Cemented stem, n (%)	11 (11.0) [b]	7 (12.5) [c]	0.560
Dislocation after reimplantation, n (%)	8 (8.0) [b]	3 (13.6) [d]	0.316
Infection eradication after reimplantation, n (%)	92 (92.0) [b]	19 (86.4) [d]	0.401

[a]n=49 after excluding nine hips with incomplete interim radiographs

[b]n=100 after excluding three hips with permanent spacers and four hips with permanent resection

[c]n=56 after excluding two hips with permanent spacers

[d]n=22 after excluding seven hips with incomplete postoperative follow-up radiographs and 27 hips with follow-up <2 years

Supplementary Table S4. Univariate analysis of risk factors associated with spacer dislocation in n-MS spacers

Variables	Stable joint (n=97)	Joint dislocation (n=10)	Odds ratio (95% CI)	P-value
Age ≥60 years, n (%)	53 (54.6)	6 (60.0)	1.16 (0.22–3.91)	0.609
Female, n (%)	47 (48.5)	6 (60.0)	3.56 (1.71–12.7)	0.053
Body mass index ≥25.0 kg/m^2, n (%)	45 (46.4)	4 (40.0)	0.85 (0.36–2.06)	0.181
Right laterality, n (%)	50 (51.5)	5 (50.0)	0.96 (0.45–3.01)	0.584
Current/ex-smokers, n (%)	38 (38.0)	4 (40.0)	1.04 (0.32–3.77)	0.593
Insurance status				
Insured, n (%)	87 (89.7)	9 (90.0)	1.01 (0.16–3.61)	0.662
Any form of Medicaid, n (%)	10 (10.3)	1 (10.0)	0.98 (0.30–2.58)	0.772
Uninsured, n (%)	0 (0.0)	0 (0.0)	1.00 (1.00–1.00)	0.999
Socioeconomic status				
Low, n (%)	45 (46.4)	4 (40.0)	0.89 (0.31–2.38)	0.608
Middle, n (%)	28 (28.9)	4 (40.0)	3.87 (0.76–14.92)	**0.049** [c]
High, n (%)	24 (24.7)	2 (20.0)	0.91 (0.17–2.71)	0.667
Charlson comorbidity index				
0–2, n (%)	52 (53.6)	5 (50.0)	0.94 (0.31–3.81)	0.497
3+, n (%)	45 (46.4)	5 (50.0)	1.35 (0.57–3.45)	0.370
McPherson host grade				

Uncompromised, n (%)	19 (19.6)	3 (30.0)	4.48 (2.63–20.4)	**0.037** [c]
Compromised, n (%)	29 (29.9)	2 (20.0)	0.90 (0.31–2.08)	0.538
Significantly compromised, n (%)	49 (50.5)	5 (50.0)	0.99 (0.24–1.81)	0.952
Microorganisms				
Gram-positive species, n (%)	56 (57.7)	6 (60.0)	1.18 (0.26–3.07)	0.751
Gram-negative species, n (%)	33 (34.0)	3 (30.0)	0.91 (0.14–2.09)	0.608
Polymicrobial, n (%)	8 (8.2)	1 (10.0)	1.03 (0.22–3.13)	0.643
Preoperative acetabular bone defects				
Paprosky type I, n (%)	84 (86.6)	6 (60.0)	0.67 (0.07–2.13)	0.139
Paprosky type II, n (%)	13 (13.4)	4 (40.0)	5.93 (1.53–24.8)	**0.030** [c]
Paprosky type III, n (%)	0 (0)	0 (0)	1.00 (1.00–1.00)	0.999
Underwent arthrotomy history	19 (19.6)	3 (30.0)	2.14 (0.26–6.81)	0.083
Surgical time ≥150 min, n (%)	61 (62.9)	6 (60.0)	0.98 (0.34–2.04)	0.615
Surgical blood loss ≥600 mL, n (%)	48 (49.5)	5 (50.0)	1.01 (0.24–2.10)	0.931
Preoperative hip parameters				
Femoral head diameter ≤44 mm, n (%)	39 (40.2)	3 (30.0)	0.85 (0.14–2.73)	0.504
Femoral neck length ≤42 mm, n (%)	56 (57.7)	5 (50.0)	0.95 (0.17–2.16)	0.691
Neck-shaft angle ≤130 degrees, n (%)	48 (49.5)	4 (40.0)	0.93 (0.20–2.27)	0.640
Femoral offset ≤34 mm, n (%)	31 (32.0)	2 (20.0)	0.77 (0.07–2.26)	0.176

Leg-length discrepancy ≥-3 mm, n (%)	78 (80.4)	7 (70.0)	0.88 (0.04–2.62)	0.517
Postoperative hip parameters				
Femoral head diameter ≤44 mm, n (%)	50 (51.5)	8 (80.0)	4.13 (1.47–19.6)	**0.044**[c]
Femoral neck length ≤42 mm, n (%)	72 (74.2)	8 (80.0)	1.39 (0.71–4.11)	0.681
Neck-shaft angle ≤130 degrees, n (%)	78 (80.4)	10 (100.0)	2.13 (0.53–7.39)	0.077
Femoral offset ≤34 mm, n (%)	74 (76.3)	8 (80.0)	1.06 (0.16–2.42)	0.501
Leg-length discrepancy ≥-3 mm, n (%)	57 (58.8)	7 (70.0)	2.11 (0.10–7.81)	0.094
Δhip parameters[a]				
Under-restored femoral head diameter (≥3 mm), n (%)	21 (21.6)	10 (100.0)	13.8 (3.74–84.1)	**<0.001** [c]
Under-restored femoral neck length (≥3 mm), n (%)	42 (43.3)	3 (30.0)	0.65 (0.13–1.82)	0.163
Under-restored neck-shaft angle (≥3 degree), n (%)	45 (46.4)	4 (40.0)	0.97 (0.12–2.36)	0.635
Under-restored femoral offset (≥3 mm), n (%)	32 (33.0)	8 (80.0)	10.4 (2.67–71.5)	**0.002**[c]
Under-restored leg-length discrepancy (≥3 mm), n (%)	61 (62.9)	7 (70.0)	1.48 (0.26–5.06)	0.441
Surgical volume ≤6 resection arthroplasties/year, n (%)	35 (36.1)	8 (80.0)	8.12 (2.03–66.4)	**0.004** [c]
Interim period[b], (≥12 weeks), n (%)	47 (48.5)	5 (50.0)	1.01 (0.04–2.38)	0.917
Follow-up period, (≥24 months), n (%)	100 (100.0)	100 (100.0)	1 (1.00–1.00)	0.999

n-MS, non-medullary sparing

[a]The spacer hip compared to the preoperative ipsilateral hip.

[b]Three hips with permanent spacers and four with permanent resection were excluded.

[c]Bold font indicates significant differences (P<0.05).
